# 2-Methyl-*N*-[1-(1*H*-pyrrol-2-yl)ethyl­idene]aniline

**DOI:** 10.1107/S1600536812045849

**Published:** 2012-11-10

**Authors:** Bi-yun Su, Wen-Long Qin, Long Jiao, Jia-Xiang Wang

**Affiliations:** a College of Chemistry and Chemical Engineering, Xi’an ShiYou University, Xi’an, Shaanxi 710065, People’s Republic of China; b College of Petroleum Engineering, Xi’an ShiYou University, Xi’an, Shaanxi 710065, People’s Republic of China

## Abstract

There are two independent mol­ecules in the asymmetric unit of the title compound, C_13_H_14_N_2_, in which the dihedral angles formed by the pyrrole and benzene rings are 83.63 (8) and 87.84 (8)°. In the crystal, mol­ecules are linked *via* pairs of N—H⋯N hydrogen bonds, forming inversion dimers, which are further connected by C—H⋯π inter­actions.

## Related literature

For general background to the imino­pyrrole unit, see: Britovsek *et al.* (2003 ▶); Dawson *et al.* (2000 ▶); Kazushi & Hayato (2005 ▶). For pyrrole monoimine, see: He *et al.* (2009 ▶); Su *et al.* (2009*a* ▶,*b* ▶).
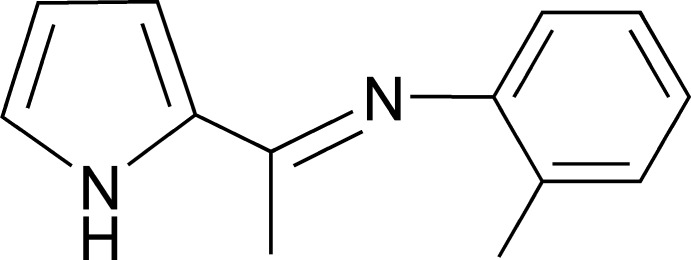


## Experimental

### 

#### Crystal data

C_13_H_14_N_2_
*M* *_r_* = 198.26
Triclinic, 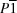

*a* = 10.120 (2) Å
*b* = 10.400 (3) Å
*c* = 11.726 (3) Å
α = 79.138 (4)°
β = 67.021 (4)°
γ = 88.154 (4)°
*V* = 1114.7 (5) Å^3^
*Z* = 4
Mo *K*α radiation
μ = 0.07 mm^−1^
*T* = 296 K
0.38 × 0.29 × 0.17 mm

#### Data collection

Bruker APEXII CCD diffractometer
Absorption correction: multi-scan (*SADABS*; Bruker, 2008 ▶) *T*_min_ = 0.973, *T*_max_ = 0.988
5729 measured reflections
3981 independent reflections
2530 reflections with *I* > 2σ(*I*)
*R*_int_ = 0.022

#### Refinement

*R*[*F* ^2^ > 2σ(*F* ^2^)] = 0.051
*wR*(*F* ^2^) = 0.149
*S* = 1.03
3981 reflections
276 parameters
2 restraints
H-atom parameters constrained
Δρ_max_ = 0.19 e Å^−3^
Δρ_min_ = −0.16 e Å^−3^

### 

Data collection: *APEX2* (Bruker,2008 ▶); cell refinement: *SAINT* (Bruker,2008 ▶); data reduction: *SAINT*; program(s) used to solve structure: *SHELXS97* (Sheldrick, 2008 ▶); program(s) used to refine structure: *SHELXL97* (Sheldrick, 2008 ▶); molecular graphics: *SHELXTL* (Sheldrick, 2008 ▶); software used to prepare material for publication: *publCIF* (Westrip, 2010 ▶).

## Supplementary Material

Crystal structure: contains datablock(s) I, global. DOI: 10.1107/S1600536812045849/lx2269sup1.cif

Structure factors: contains datablock(s) I. DOI: 10.1107/S1600536812045849/lx2269Isup2.hkl

Supplementary material file. DOI: 10.1107/S1600536812045849/lx2269Isup3.cml

Additional supplementary materials:  crystallographic information; 3D view; checkCIF report

## Figures and Tables

**Table 1:** Hydrogen-bond geometry (Å, °) *Cg*1 and *Cg*2 are the centroids of the C7–C12 and C20–C25 rings, respectively.

*D*—H⋯*A*	*D*—H	H⋯*A*	*D*⋯*A*	*D*—H⋯*A*
N1—H1⋯N2^i^	0.86	2.27	3.070 (3)	154
N3—H3⋯N4^ii^	0.86	2.30	3.108 (2)	156
C1—H1*A*⋯*Cg*1^iii^	0.93	2.70	3.488 (4)	143
C14—H14⋯*Cg*2^iv^	0.93	2.75	3.531 (3)	142

Symmetry codes: (i) 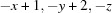; (ii) 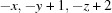; (iii) 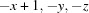; (iv) 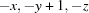.

**Fig. 1.**
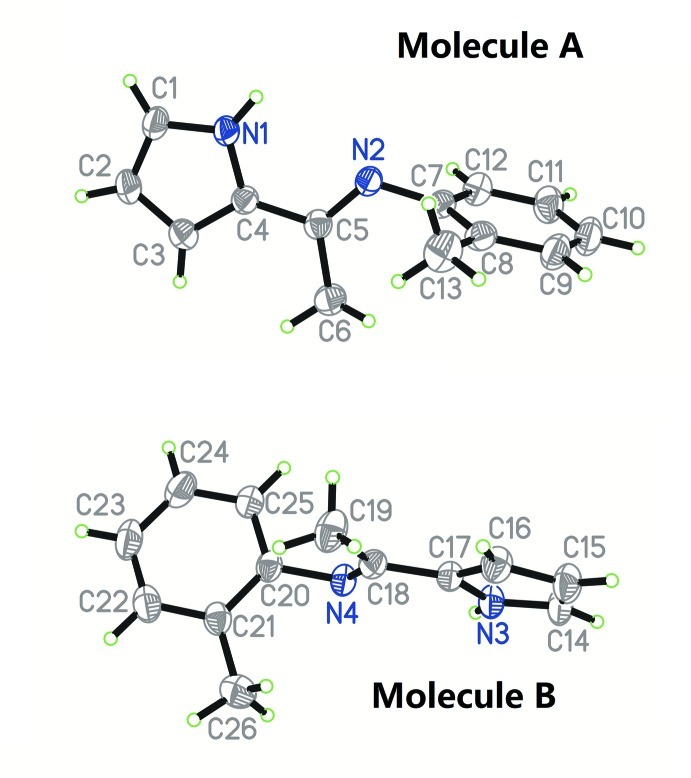
Two independent molecules in the asymmetric unit of the title compound showing the atomic numbering scheme. Displacement ellipsoids are drawn at 30% probability level. H atoms are presented as a small spheres of arbitrary radius.

**Fig. 2.**
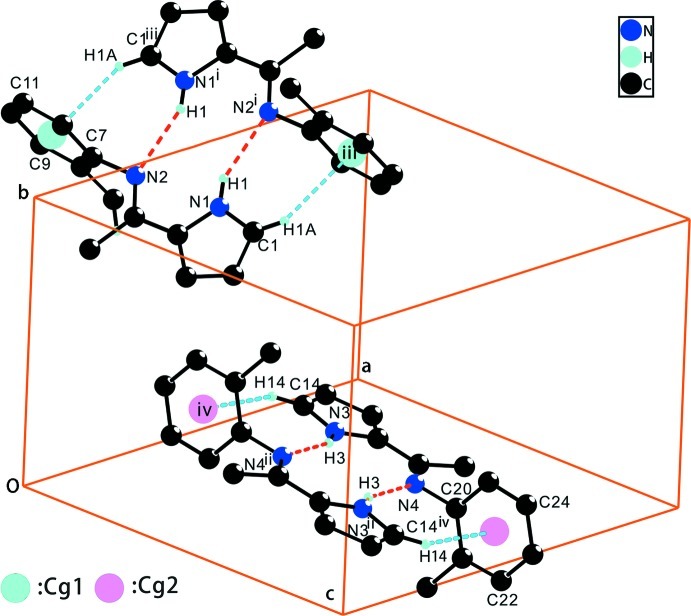
A view of N–H···N and C–H···π interactions (dotted lines) in the crystal structure of the title compound. H atoms non-participating in hydrogen-bonding were omitted for clarity. [Symmetry codes: (i) - x + 1, - y + 2, - z; (ii) - x, - y + 1, - z + 2; (iii) - x + 1, - y, - z; (iv) - x, - y + 1, - z.]

## supplementary crystallographic information

### Comment

Nitrogen-based ligands containing an iminopyrrole unit have recently drawn much attention because of their flexible complexation to transition metals (Kazushi & Hayato, 2005). Being the typical kind of iminopyrrole units, bis(imino)pyridine incorporated late transition metal catalysts have been investigated due to their antioxidant properties and outstanding activities for olefin polymerization. As a five-membered analogue of the pyridine ring (He *et al.*, 2009), pyrrole has been frequently introduced into the skeleton of bis(imino)pyridine ligands to design new ligands and corresponding metal complexes (Britovsek *et al.*, 2003; Dawson *et al.*, 2000). As a part of our ongoing studies on mono(imino)pyrrole ligands (Su *et al.*, 2009**a*,b*), we report herein the crystal structure of the title compound, which crystallizes with two unique molecules, A & B, in the asymmetric unit (Fig. 1).

In the title compound, the dihedral angles formed by the pyrrole and benzene rings are 83.63 (8)° in A and 87.84 (8)° in B, respectively. The imino N–C bond lengths in the two molecules are slightly different [1.275 (3) Å for A, 1.278 (2) Å for B, respectively] and indicate C═N character. Although the two molecules in the asymmetric unit are similar, some minor differences in corresponding bond angles are evident, most notably C–N(imino)–C of 119.54 (18)° and 119.24 (17)°, for A and B, respectively. In the crystal structure, molecules are linked *via* pairs of N–H···N hydrogen bonds (Fig. 2 & Table 1), forming inversion dimers. These dimers are connected by C–H···π interactions (Fig. 2 & Table 1, *Cg*1 and *Cg*2 are the centroids of benzene rings in the molecule A and B, respectively).

### Experimental

The reagents 2-acetyl pyrrole (0.1528 g, 1.40 mmol), *o*-toluidine (0.3000 g, 2.80 mmol) were placed in a 50-ml flask, after a few drops of acetic acid was added in, the mixture was subjected to radiation in a 800 W microwave oven for 3 min and 2 min on a medium-heat setting. The reaction was monitored by TLC, and the crude product was purified by silica gel column chromatography (eluant: petroleum ether/ethyl acetate, 5:1 *v/v*), the colourless crystals of the title compound were at last obtained by recrystallization from ethanol (yield 0.1136 g, 40.91%). m.p. 396.5-398.9 K. Plate like colourless single crystals used in X-ray diffraction studies were grown in the mixture solvents of water and ethanol.

### Refinement

All H atoms were placed at calculated positions and refined as riding, with C–H = 0.93-0.96 Å, N–H = 0.86 Å, and with *U*_iso_(H) = 1.2 *U*_eq_(C, N) or 1.5 *U*_eq_(C) for methyl H atoms. The positions of methyl hydrogens were optimized rotationally.

### Crystal data

**Table:**

C_13_H_14_N_2_	*Z* = 4
*M**_r_* = 198.26	*F*(000) = 424
Triclinic, *P*1	*D*_x_ = 1.181 Mg m^−^^3^
Hall symbol: -P 1	Melting point = 396.5–398.9 K
*a* = 10.120 (2) Å	Mo *K*α radiation, λ = 0.71073 Å
*b* = 10.400 (3) Å	Cell parameters from 1216 reflections
*c* = 11.726 (3) Å	θ = 2.3–25.2°
α = 79.138 (4)°	µ = 0.07 mm^−^^1^
β = 67.021 (4)°	*T* = 296 K
γ = 88.154 (4)°	Block, colourless
*V* = 1114.7 (5) Å^3^	0.38 × 0.29 × 0.17 mm

### Data collection

**Table:**

Bruker APEXII CCD diffractometer	3981 independent reflections
Radiation source: fine-focus sealed tube	2530 reflections with *I* > 2σ(*I*)
Graphite monochromator	*R*_int_ = 0.022
φ and ω scans	θ_max_ = 25.2°, θ_min_ = 2.3°
Absorption correction: multi-scan (*SADABS*; Bruker, 2008)	*h* = −9→12
*T*_min_ = 0.973, *T*_max_ = 0.988	*k* = −12→12
5729 measured reflections	*l* = −10→14

### Refinement

**Table:**

Refinement on *F*^2^	Secondary atom site location: difference Fourier map
Least-squares matrix: full	Hydrogen site location: inferred from neighbouring sites
*R*[*F*^2^ > 2σ(*F*^2^)] = 0.051	H-atom parameters constrained
*wR*(*F*^2^) = 0.149	*w* = 1/[σ^2^(*F*_o_^2^) + (0.0703*P*)^2^] where *P* = (*F*_o_^2^ + 2*F*_c_^2^)/3
*S* = 1.03	(Δ/σ)_max_ < 0.001
3981 reflections	Δρ_max_ = 0.19 e Å^−^^3^
276 parameters	Δρ_min_ = −0.16 e Å^−^^3^
2 restraints	Extinction correction: *SHELXL97* (Sheldrick, 2008), Fc^*^=kFc[1+0.001xFc^2^λ^3^/sin(2θ)]^-1/4^
Primary atom site location: structure-invariant direct methods	Extinction coefficient: 0.012 (3)

### Special details

**Table:**

Experimental. The purity and the composition of the compound were checked and characterized by Infrared spectrum, ^1^H NMR spectrum, mass spectrum, as well as elemental analysis. IR (KBr): ν_C__═__N_ 1645 cm^-1^. ^1^H NMR (400MHz, CDCl_3_): *δ* 7.36 (d, 1H, benzene ring aromatic H), 7.15 (t, 2H, benzene ring aromatic H), 7.88 (t, 1H, benzene ring aromatic H), 6.90 (d, 1H,pyrrole ring aromatic H), 6.71 (t, 1H, pyrrole ring aromatic H), 6.34 (d, 1H,pyrrole ring aromatic H), 2.48 (s, 3H, phenyl-CH_3_), 2.25 (s, 3H,-N═C(CH_3_)-). MS (EI): m/*z* 197 (*M^+^*). Anal. Calcd. for C_13_H_14_N_2_: C, 78.75; H, 7.12; N, 14.13. Found: C, 78.22; H, 6.95; N,14.68.
Geometry. All e.s.d.'s (except the e.s.d. in the dihedral angle between two l.s. planes) are estimated using the full covariance matrix. The cell e.s.d.'s are taken into account individually in the estimation of e.s.d.'s in distances, angles and torsion angles; correlations between e.s.d.'s in cell parameters are only used when they are defined by crystal symmetry. An approximate (isotropic) treatment of cell e.s.d.'s is used for estimating e.s.d.'s involving l.s. planes.
Refinement. Refinement of F^2^ against ALL reflections. The weighted R-factor wR and goodness of fit S are based on F^2^, conventional R-factors R are based on F, with F set to zero for negative F^2^. The threshold expression of F^2^ > 2sigma(F^2^) is used only for calculating R-factors(gt) etc. and is not relevant to the choice of reflections for refinement. R-factors based on F^2^ are statistically about twice as large as those based on F, and R- factors based on ALL data will be even larger.

### Fractional atomic coordinates and isotropic or equivalent isotropic displacement parameters (Å^2^)

**Table:**

	*x*	*y*	*z*	*U*_iso_*/*U*_eq_	
N1	0.37284 (18)	0.91215 (17)	0.19040 (14)	0.0510 (5)	
H1	0.4426	0.9579	0.1289	0.061*	
N2	0.34013 (18)	0.93166 (17)	−0.03844 (14)	0.0507 (5)	
N3	0.14266 (18)	0.49423 (17)	0.80750 (14)	0.0541 (5)	
H3	0.0662	0.5111	0.8678	0.065*	
N4	0.16015 (18)	0.41253 (17)	1.04284 (14)	0.0498 (5)	
C1	0.3590 (3)	0.8857 (2)	0.31132 (18)	0.0626 (6)	
H1A	0.4226	0.9137	0.3422	0.075*	
C2	0.2371 (3)	0.8118 (3)	0.3801 (2)	0.0689 (7)	
H2	0.2016	0.7798	0.4666	0.083*	
C3	0.1742 (3)	0.7922 (2)	0.2987 (2)	0.0633 (6)	
H3A	0.0888	0.7445	0.3208	0.076*	
C4	0.2595 (2)	0.8553 (2)	0.18026 (17)	0.0473 (5)	
C5	0.2429 (2)	0.8691 (2)	0.06202 (19)	0.0491 (5)	
C6	0.1083 (2)	0.8091 (3)	0.0660 (2)	0.0724 (7)	
H6A	0.0392	0.8752	0.0691	0.109*	
H6B	0.0696	0.7412	0.1396	0.109*	
H6C	0.1299	0.7724	−0.0082	0.109*	
C7	0.3199 (2)	0.95209 (18)	−0.15330 (18)	0.0502 (5)	
C8	0.3676 (2)	0.8647 (2)	−0.2347 (2)	0.0562 (6)	
C9	0.3553 (3)	0.8960 (3)	−0.3496 (2)	0.0681 (7)	
H9	0.3870	0.8379	−0.4052	0.082*	
C10	0.2981 (3)	1.0095 (3)	−0.3841 (2)	0.0753 (8)	
H10	0.2898	1.0276	−0.4617	0.090*	
C11	0.2530 (3)	1.0965 (3)	−0.3044 (2)	0.0712 (7)	
H11	0.2154	1.1749	−0.3284	0.085*	
C12	0.2627 (2)	1.06875 (19)	−0.18953 (19)	0.0589 (6)	
H12	0.2311	1.1280	−0.1352	0.071*	
C13	0.4357 (3)	0.7415 (3)	−0.2007 (2)	0.0795 (8)	
H13A	0.4550	0.6895	−0.2645	0.119*	
H13B	0.3716	0.6927	−0.1209	0.119*	
H13C	0.5240	0.7633	−0.1949	0.119*	
C14	0.1659 (3)	0.5221 (3)	0.68454 (19)	0.0672 (7)	
H14	0.1023	0.5622	0.6508	0.081*	
C15	0.2975 (3)	0.4818 (3)	0.6179 (2)	0.0707 (7)	
H15	0.3410	0.4898	0.5307	0.085*	
C16	0.3551 (2)	0.4261 (2)	0.70452 (19)	0.0630 (6)	
H16	0.4442	0.3890	0.6856	0.076*	
C17	0.2584 (2)	0.4353 (2)	0.82227 (17)	0.0462 (5)	
C18	0.2667 (2)	0.3960 (2)	0.94336 (19)	0.0475 (5)	
C19	0.4037 (2)	0.3381 (3)	0.9440 (2)	0.0737 (8)	
H19A	0.4699	0.4068	0.9358	0.111*	
H19B	0.4450	0.2930	0.8747	0.111*	
H19C	0.3843	0.2775	1.0219	0.111*	
C20	0.1708 (2)	0.37695 (19)	1.16170 (18)	0.0489 (5)	
C21	0.1223 (2)	0.2549 (2)	1.23561 (19)	0.0544 (6)	
C22	0.1255 (3)	0.2295 (3)	1.3541 (2)	0.0698 (7)	
H22	0.0941	0.1472	1.4047	0.084*	
C23	0.1739 (3)	0.3221 (3)	1.3993 (2)	0.0791 (8)	
H23	0.1750	0.3026	1.4796	0.095*	
C24	0.2206 (3)	0.4433 (3)	1.3258 (2)	0.0766 (8)	
H24	0.2528	0.5068	1.3564	0.092*	
C25	0.2199 (2)	0.4712 (2)	1.2078 (2)	0.0611 (6)	
H25	0.2524	0.5535	1.1577	0.073*	
C26	0.0651 (3)	0.1542 (3)	1.1890 (2)	0.0790 (8)	
H26A	−0.0239	0.1815	1.1831	0.119*	
H26B	0.0491	0.0719	1.2467	0.119*	
H26C	0.1333	0.1444	1.1073	0.119*	

### Atomic displacement parameters (Å^2^)

**Table:**

	*U*^11^	*U*^22^	*U*^33^	*U*^12^	*U*^13^	*U*^23^
N1	0.0470 (10)	0.0660 (12)	0.0362 (9)	−0.0026 (9)	−0.0132 (8)	−0.0066 (8)
N2	0.0493 (11)	0.0624 (12)	0.0430 (9)	−0.0007 (9)	−0.0207 (8)	−0.0098 (8)
N3	0.0512 (11)	0.0680 (12)	0.0406 (9)	0.0081 (9)	−0.0185 (8)	−0.0047 (8)
N4	0.0497 (11)	0.0586 (11)	0.0420 (9)	0.0077 (8)	−0.0210 (8)	−0.0060 (8)
C1	0.0668 (16)	0.0836 (18)	0.0405 (12)	−0.0020 (13)	−0.0240 (11)	−0.0119 (11)
C2	0.0707 (17)	0.0882 (19)	0.0382 (12)	−0.0102 (14)	−0.0159 (12)	0.0010 (12)
C3	0.0568 (14)	0.0754 (17)	0.0507 (13)	−0.0094 (12)	−0.0173 (11)	−0.0019 (11)
C4	0.0446 (12)	0.0539 (13)	0.0447 (11)	0.0020 (10)	−0.0188 (10)	−0.0092 (10)
C5	0.0465 (12)	0.0529 (13)	0.0485 (12)	0.0058 (10)	−0.0204 (10)	−0.0081 (10)
C6	0.0546 (15)	0.099 (2)	0.0655 (15)	−0.0131 (13)	−0.0301 (12)	−0.0033 (14)
C7	0.0441 (12)	0.0617 (14)	0.0469 (11)	−0.0025 (10)	−0.0198 (10)	−0.0101 (10)
C8	0.0521 (13)	0.0617 (15)	0.0578 (13)	−0.0028 (11)	−0.0231 (11)	−0.0140 (11)
C9	0.0709 (16)	0.089 (2)	0.0518 (13)	0.0025 (14)	−0.0272 (12)	−0.0235 (13)
C10	0.0771 (18)	0.106 (2)	0.0484 (13)	0.0019 (16)	−0.0318 (13)	−0.0121 (14)
C11	0.0731 (17)	0.0820 (18)	0.0615 (14)	0.0102 (14)	−0.0348 (13)	−0.0030 (13)
C12	0.0620 (15)	0.0655 (16)	0.0542 (13)	0.0093 (12)	−0.0272 (11)	−0.0147 (11)
C13	0.093 (2)	0.0716 (18)	0.0840 (17)	0.0206 (15)	−0.0420 (15)	−0.0266 (14)
C14	0.0681 (16)	0.0907 (19)	0.0418 (12)	0.0079 (14)	−0.0256 (11)	−0.0023 (12)
C15	0.0711 (17)	0.096 (2)	0.0401 (12)	0.0057 (14)	−0.0187 (12)	−0.0096 (12)
C16	0.0558 (14)	0.0799 (17)	0.0514 (13)	0.0084 (12)	−0.0185 (11)	−0.0143 (12)
C17	0.0457 (12)	0.0503 (13)	0.0423 (11)	0.0044 (10)	−0.0181 (9)	−0.0068 (9)
C18	0.0471 (12)	0.0495 (13)	0.0497 (12)	0.0050 (10)	−0.0232 (10)	−0.0097 (10)
C19	0.0567 (15)	0.106 (2)	0.0635 (14)	0.0267 (14)	−0.0295 (12)	−0.0191 (14)
C20	0.0426 (12)	0.0603 (14)	0.0442 (11)	0.0097 (10)	−0.0195 (9)	−0.0069 (10)
C21	0.0506 (13)	0.0588 (15)	0.0515 (13)	0.0090 (11)	−0.0210 (10)	−0.0041 (11)
C22	0.0703 (17)	0.0769 (18)	0.0546 (14)	0.0055 (14)	−0.0248 (12)	0.0057 (13)
C23	0.0847 (19)	0.106 (2)	0.0515 (14)	0.0099 (17)	−0.0372 (14)	−0.0048 (15)
C24	0.090 (2)	0.089 (2)	0.0695 (16)	0.0069 (16)	−0.0487 (15)	−0.0221 (15)
C25	0.0683 (16)	0.0643 (16)	0.0566 (13)	0.0023 (12)	−0.0324 (12)	−0.0078 (11)
C26	0.092 (2)	0.0623 (17)	0.0823 (17)	−0.0068 (14)	−0.0358 (15)	−0.0085 (13)

### Geometric parameters (Å, º)

**Table:**

N1—C1	1.344 (2)	C11—H11	0.9300
N1—C4	1.362 (3)	C12—H12	0.9300
N1—H1	0.8600	C13—H13A	0.9600
N2—C5	1.275 (3)	C13—H13B	0.9600
N2—C7	1.416 (2)	C13—H13C	0.9600
N3—C14	1.342 (2)	C14—C15	1.355 (3)
N3—C17	1.360 (2)	C14—H14	0.9300
N3—H3	0.8600	C15—C16	1.388 (3)
N4—C18	1.278 (2)	C15—H15	0.9300
N4—C20	1.418 (2)	C16—C17	1.364 (3)
C1—C2	1.347 (3)	C16—H16	0.9300
C1—H1A	0.9300	C17—C18	1.436 (3)
C2—C3	1.383 (3)	C18—C19	1.496 (3)
C2—H2	0.9300	C19—H19A	0.9600
C3—C4	1.364 (3)	C19—H19B	0.9600
C3—H3A	0.9300	C19—H19C	0.9600
C4—C5	1.441 (3)	C20—C21	1.377 (3)
C5—C6	1.497 (3)	C20—C25	1.3927 (10)
C6—H6A	0.9600	C21—C22	1.378 (3)
C6—H6B	0.9600	C21—C26	1.494 (3)
C6—H6C	0.9600	C22—C23	1.369 (4)
C7—C8	1.382 (3)	C22—H22	0.9300
C7—C12	1.3925 (10)	C23—C24	1.366 (4)
C8—C9	1.378 (3)	C23—H23	0.9300
C8—C13	1.497 (3)	C24—C25	1.362 (3)
C9—C10	1.360 (3)	C24—H24	0.9300
C9—H9	0.9300	C25—H25	0.9300
C10—C11	1.364 (3)	C26—H26A	0.9600
C10—H10	0.9300	C26—H26B	0.9600
C11—C12	1.366 (3)	C26—H26C	0.9600
			
C1—N1—C4	109.55 (18)	H13A—C13—H13B	109.5
C1—N1—H1	125.2	C8—C13—H13C	109.5
C4—N1—H1	125.2	H13A—C13—H13C	109.5
C5—N2—C7	119.54 (18)	H13B—C13—H13C	109.5
C14—N3—C17	109.63 (17)	N3—C14—C15	108.52 (19)
C14—N3—H3	125.2	N3—C14—H14	125.7
C17—N3—H3	125.2	C15—C14—H14	125.7
C18—N4—C20	119.24 (17)	C14—C15—C16	106.91 (19)
N1—C1—C2	108.3 (2)	C14—C15—H15	126.5
N1—C1—H1A	125.9	C16—C15—H15	126.5
C2—C1—H1A	125.9	C17—C16—C15	108.2 (2)
C1—C2—C3	107.55 (19)	C17—C16—H16	125.9
C1—C2—H2	126.2	C15—C16—H16	125.9
C3—C2—H2	126.2	N3—C17—C16	106.70 (17)
C4—C3—C2	108.0 (2)	N3—C17—C18	122.69 (17)
C4—C3—H3A	126.0	C16—C17—C18	130.61 (19)
C2—C3—H3A	126.0	N4—C18—C17	119.61 (18)
N1—C4—C3	106.62 (18)	N4—C18—C19	123.84 (18)
N1—C4—C5	122.36 (18)	C17—C18—C19	116.54 (18)
C3—C4—C5	131.0 (2)	C18—C19—H19A	109.5
N2—C5—C4	119.52 (19)	C18—C19—H19B	109.5
N2—C5—C6	123.74 (18)	H19A—C19—H19B	109.5
C4—C5—C6	116.74 (19)	C18—C19—H19C	109.5
C5—C6—H6A	109.5	H19A—C19—H19C	109.5
C5—C6—H6B	109.5	H19B—C19—H19C	109.5
H6A—C6—H6B	109.5	C21—C20—C25	120.03 (19)
C5—C6—H6C	109.5	C21—C20—N4	120.84 (17)
H6A—C6—H6C	109.5	C25—C20—N4	118.90 (19)
H6B—C6—H6C	109.5	C20—C21—C22	118.1 (2)
C8—C7—C12	119.60 (18)	C20—C21—C26	120.66 (19)
C8—C7—N2	121.35 (16)	C22—C21—C26	121.2 (2)
C12—C7—N2	118.78 (17)	C23—C22—C21	121.8 (2)
C9—C8—C7	118.4 (2)	C23—C22—H22	119.1
C9—C8—C13	120.9 (2)	C21—C22—H22	119.1
C7—C8—C13	120.72 (19)	C24—C23—C22	119.7 (2)
C10—C9—C8	121.8 (2)	C24—C23—H23	120.1
C10—C9—H9	119.1	C22—C23—H23	120.1
C8—C9—H9	119.1	C25—C24—C23	120.0 (2)
C9—C10—C11	119.8 (2)	C25—C24—H24	120.0
C9—C10—H10	120.1	C23—C24—H24	120.0
C11—C10—H10	120.1	C24—C25—C20	120.4 (2)
C10—C11—C12	120.1 (2)	C24—C25—H25	119.8
C10—C11—H11	119.9	C20—C25—H25	119.8
C12—C11—H11	119.9	C21—C26—H26A	109.5
C11—C12—C7	120.3 (2)	C21—C26—H26B	109.5
C11—C12—H12	119.8	H26A—C26—H26B	109.5
C7—C12—H12	119.8	C21—C26—H26C	109.5
C8—C13—H13A	109.5	H26A—C26—H26C	109.5
C8—C13—H13B	109.5	H26B—C26—H26C	109.5
			
C4—N1—C1—C2	0.0 (3)	C17—N3—C14—C15	−0.2 (3)
N1—C1—C2—C3	0.0 (3)	N3—C14—C15—C16	0.7 (3)
C1—C2—C3—C4	−0.1 (3)	C14—C15—C16—C17	−0.9 (3)
C1—N1—C4—C3	−0.1 (2)	C14—N3—C17—C16	−0.4 (3)
C1—N1—C4—C5	178.53 (18)	C14—N3—C17—C18	178.8 (2)
C2—C3—C4—N1	0.1 (3)	C15—C16—C17—N3	0.8 (3)
C2—C3—C4—C5	−178.4 (2)	C15—C16—C17—C18	−178.3 (2)
C7—N2—C5—C4	−176.19 (16)	C20—N4—C18—C17	−178.17 (18)
C7—N2—C5—C6	2.9 (3)	C20—N4—C18—C19	1.2 (3)
N1—C4—C5—N2	3.2 (3)	N3—C17—C18—N4	2.2 (3)
C3—C4—C5—N2	−178.6 (2)	C16—C17—C18—N4	−178.8 (2)
N1—C4—C5—C6	−176.01 (19)	N3—C17—C18—C19	−177.2 (2)
C3—C4—C5—C6	2.3 (3)	C16—C17—C18—C19	1.8 (4)
C5—N2—C7—C8	−91.5 (2)	C18—N4—C20—C21	−93.6 (2)
C5—N2—C7—C12	94.5 (3)	C18—N4—C20—C25	91.8 (2)
C12—C7—C8—C9	−0.7 (3)	C25—C20—C21—C22	−0.7 (3)
N2—C7—C8—C9	−174.6 (2)	N4—C20—C21—C22	−175.18 (18)
C12—C7—C8—C13	177.2 (2)	C25—C20—C21—C26	178.1 (2)
N2—C7—C8—C13	3.2 (3)	N4—C20—C21—C26	3.6 (3)
C7—C8—C9—C10	0.0 (4)	C20—C21—C22—C23	0.7 (3)
C13—C8—C9—C10	−177.8 (3)	C26—C21—C22—C23	−178.1 (2)
C8—C9—C10—C11	0.9 (4)	C21—C22—C23—C24	−0.1 (4)
C9—C10—C11—C12	−1.2 (4)	C22—C23—C24—C25	−0.5 (4)
C10—C11—C12—C7	0.5 (4)	C23—C24—C25—C20	0.5 (4)
C8—C7—C12—C11	0.4 (3)	C21—C20—C25—C24	0.1 (3)
N2—C7—C12—C11	174.5 (2)	N4—C20—C25—C24	174.74 (19)

### Hydrogen-bond geometry (Å, º)

Cg1 and Cg2 are the centroids of the C7–C12 and C20–C25 rings, respectively.

**Table:**

*D*—H···*A*	*D*—H	H···*A*	*D*···*A*	*D*—H···*A*
N1—H1···N2^i^	0.86	2.27	3.070 (3)	154
N3—H3···N4^ii^	0.86	2.30	3.108 (2)	156
C1—H1*A*···*Cg*1^iii^	0.93	2.70	3.488 (4)	143
C14—H14···*Cg*2^iv^	0.93	2.75	3.531 (3)	142

Symmetry codes: (i) −*x*+1, −*y*+2, −*z*; (ii) −*x*, −*y*+1, −*z*+2; (iii) −*x*+1, −*y*, −*z*; (iv) −*x*, −*y*+1, −*z*.
